# Weight Change and the Onset of Cardiovascular Diseases: Emulating Trials Using Electronic Health Records

**DOI:** 10.1097/EDE.0000000000001393

**Published:** 2021-07-28

**Authors:** Michail Katsoulis, Bianca D. Stavola, Karla Diaz-Ordaz, Manuel Gomes, Alvina Lai, Pagona Lagiou, Goya Wannamethee, Konstantinos Tsilidis, R. Thomas Lumbers, Spiros Denaxas, Amitava Banerjee, Constantinos A. Parisinos, Rachel Batterham, Riyaz Patel, Claudia Langenberg, Harry Hemingway

**Affiliations:** From the a Institute of Health Informatics, University College London (UCL), London, United Kingdom; b Health Data Research UK London, UCL, London, United Kingdom; c Great Ormond Street Institute of Child Health, UCL, London, United Kingdom; d Medical Statistics Department, London School of Hygiene and Tropical Medicine, London, United Kingdom; e Institute of Epidemiology & Health Care, UCL, London, United Kingdom; f Department of Hygiene, Epidemiology and Medical Statistics, School of Medicine, National and Kapodistrian University of Athens, Athens, Greece; g Department of Epidemiology, Harvard T.H. Chan School of Public Health, Boston, MA; h Department of Epidemiology and Biostatistics, Imperial College London, London, United Kingdom; i Department of Hygiene and Epidemiology, University of Ioannina School of Medicine, Ioannina, Greece; j Bart’s Heart Centre, St Bartholomew’s Hospital, London, United Kingdom; k The Alan Turing Institute, London, United Kingdom; l The National Institute for Health Research, University College London Hospitals Biomedical Research Centre, UCL, London, United Kingdom; m British Heart Foundation Research Accelerator, UCL, London, United Kingdom; n Amrita Institute of Medical Sciences, Kochi, India; o Centre for Obesity Research, UCL, London, United Kingdom; p University College London Hospitals Bariatric Centre for Weight Management and Metabolic Surgery, London, United Kingdom; q Institute of Cardiovascular Science, UCL, London, United Kingdom; r MRC Epidemiology Unit, University of Cambridge, Cambridge, United Kingdom.

## Abstract

Cross-sectional measures of body mass index (BMI) are associated with cardiovascular disease (CVD) incidence, but less is known about whether weight change affects the risk of CVD.

**Methods:**

We estimated the effect of 2-y weight change interventions on 7-y risk of CVD (CVD death, myocardial infarction, stroke, hospitalization from coronary heart disease, and heart failure) by emulating hypothetical interventions using electronic health records. We identified 138,567 individuals with 45–69 years of age without chronic disease in England from 1998 to 2016. We performed pooled logistic regression, using inverse-probability weighting to adjust for baseline and time-varying confounders. We categorized each individual into a weight loss, maintenance, or gain group.

**Results:**

Among those of normal weight, both weight loss [risk difference (RD) vs. weight maintenance = 1.5% (0.3% to 3.0%)] and gain [RD = 1.3% (0.5% to 2.2%)] were associated with increased risk for CVD compared with weight maintenance. Among overweight individuals, we observed moderately higher risk of CVD in both the weight loss [RD = 0.7% (−0.2% to 1.7%)] and the weight gain group [RD = 0.7% (−0.1% to 1.7%)], compared with maintenance. In the obese, those losing weight showed lower risk of coronary heart disease [RD = −1.4% (−2.4% to −0.6%)] but not of stroke. When we assumed that chronic disease occurred 1–3 years before the recorded date, estimates for weight loss and gain were attenuated among overweight individuals; estimates for loss were lower among obese individuals.

**Conclusion:**

Among individuals with obesity, the weight-loss group had a lower risk of coronary heart disease but not of stroke. Weight gain was associated with increased risk of CVD across BMI groups. See video abstract at, http://links.lww.com/EDE/B838.

## Abstract 2

Supplemental Digital Content is available in the text.

## INTRODUCTION

Lifestyle^1^ and pharmacotherapy interventions^2,3^ have been shown in randomized controlled trials (RCTs) to be effective in achieving weight loss among individuals with overweight or obesity. In such high-risk patients, there is evidence that weight loss is also beneficial for cardiovascular risk factors.^4^ However, there are mixed findings on the effect of weight loss on cardiovascular disease (CVD), as some studies found no effect of weight loss on CVD,^1,5,6^ although a recent meta-analysis of trials reported moderate lower risk of CVD following weight loss.^4^ In any case, there are no RCTs of any weight-loss intervention assessing the effectiveness for primary prevention of CVDs in otherwise healthy individuals with overweight or obesity. This is particularly important as higher body mass index (BMI) is associated with the onset of cardiovascular diseases,^7^ and the prevalence of obesity, already high, is predicted to increase further.^8^

Emulating target pragmatic trials using causal inference methods in large-scale observational data may play a role in distinguishing effects of weight change in people with normal weight, overweight, and obesity.^9-12^ Observational studies of weight or BMI change are conflicting, with studies reporting variously increased risk of CVD,^13-16^ no association,^17-19^ or lower risk. Studies looking at people with severe obesity who had bariatric surgery experienced particular reductions in risk.^20^ Weight gain has been associated with increased CVD risk in some studies,^13-16^ but not others.^21^ The Nurses’ Health Study has emulated weight-loss trials (i.e., in a consented cohort), which found no relationship between weight loss and CHD.^17,18^ To our knowledge, there have been no previous emulation of weight-loss trials reporting separate effects within groups of people with normal weight, overweight, or obesity. This distinction is very important because weight loss might be more beneficial for individuals with obesity^20^ than for overweight individuals,^19^ although weight loss might have adverse effects on CVD in normal-weight individuals.^13^

In this study, we investigated hypothetical interventions using electronic health records (EHR) and assessed whether weight change affects the occurrence of CVDs. This is to our knowledge the largest study (~138 K) to estimate the effect of weight change on CVDs in individuals with 45–69 years of age and the first one to investigate weight change separately in individuals of normal weight, overweight, and obesity without chronic diseases.

## METHODS

### Data Sources

We analyzed data from individuals from the CALIBER programme in England between January 1, 1998, and June 30, 2016. CALIBER links anonymized coded EHR from three national data sources of patients across primary care (Clinical Practice Research Datalink), hospital care (Hospital Episode Statistics), and death registry (Office of National Statistics), from a large sample, representative of the population of England.^22^ Methods for the development of reproducible phenotypes and metadata have been described previously^23^ and are available online (www.caliberresearch.org/portal). The study was approved by the Independent Scientific Advisory Committee (ISAC) of the Medicines and Healthcare Products Regulatory Agency (United Kingdom).

### Covariates

Height and weight measurements were based on primary care records and measured as part of routine care. We divided our sample into three groups based on standard clinical BMI cutoffs: individuals with (i) normal weight (18.5–24.9 kg/m^2^), (ii) overweight (25–29.9 kg/m^2^), and (iii) obesity (30–39.9 kg/m^2^).

We also used information on the following variables: age at baseline, sex, region, index of multiple deprivations (IMD), ethnicity, smoking status, physical activity, diabetes, cancer (apart from nonmelanoma skin cancer), dementia, severe mental diseases, chronic kidney disease, chronic obstructive pulmonary disease, HIV, major inflammatory diseases, Parkinson’s disease, multiple sclerosis, renal failure (or initiation of dialysis), prevalence of hypertension, antihypertensive medication, number of weight measurements, and number of clinical consultations every year.

### Target Trials Specification and Emulation

We first explicitly specified the target trials of interest and then used EHR to emulate them.^9-12^ We present the protocol of the target trial, along with the protocol of the trial emulation below, in Table 1, and we provide further details in the eAppendix (Section 2); http://links.lww.com/EDE/B828.

**TABLE 1.** Specification and Emulation of Target Trials to Estimate the Effect of Weight Change on Various CVD Outcomes. Description for the three main trials in normal weight, overweight and obese individuals

	Target Trials	Emulation of Target Trials Using CALIBER Data
Eligibility criteria	Trials would enroll healthy^a^ individuals at baseline in England, aged 45–69 years old between 1998 and 2016. We would exclude participants who would have undergone bariatric surgery before baseline or who would be unhealthy^a^ before baseline. The trials will be conducted in different population groups by BMI levels (different trials for normal weight, overweight, and obese people). Baseline is defined as the date of the first BMI and weight observations, given that all eligibility criteria are met.	Same plus: (i) all participants should have measurements of smoking status. Especially for those individuals with information on both physical activity and smoking status, we selected as baseline date, the date of the first BMI, and weight observations, in which they had information on both smoking status and physical activity. (ii) emulated trial applied the exclusion criteria from chronic diseases based on observing a 12-month baseline period and(iii) excluded individuals who had ≥12 clinical consultations or measured their bodyweight ≥6 times in the primary care^b^ during the baseline period (first year)
Treatment strategies	(a) lose 3–20% of their weight each year or undergo bariatric surgery (b) maintain their weight, (weight change >−3% and <3% of bodyweight each year) (c) gain 3–20% of their weight each year These hypothetical interventions will be followed for 2 years. Individuals were free to deviate after the end of the second year. Individuals would be allowed to deviate from their assigned intervention if they developed clinically allowable reasons for deviating from assigned intervention	Same + Individuals are allowed to deviate from their assigned intervention if they have ≥12 clinical consultations or measured their body weight ≥6 times per year in the primary care^b^ during the second year
Assignment procedures	Individuals would be randomly assigned to a strategy at baseline and will be aware of the strategy to which they have been assigned.	Randomization is emulated via. adjustment for baseline covariates^c^ in the outcome regression models
Follow-up period	Starts at baseline and ends at CVD diagnosis, death, loss to follow-up (transfer out, 7 years after baseline, or administrative end of follow-up (31 June 2016), whichever occurs first.	Same
Endpoints	Primary endpoint: Composite CVD outcome (CVD deaths, nonfatal Myocardial infarction, nonfatal stroke, hospitalization from coronary heart disease, hospitalization from heart failure) Secondary endpoints: (a) Composite CHD outcome (CHD deaths, nonfatal Myocardial infarction, hospitalization from CHD) (b) Myocardial infarction, (c) fatal and nonfatal Stroke and (d) heart failure (hospitalizations or deaths), and (e) CVD deaths	Same + sensitivity analysis for (a) diabetes (positive control outcome) (b) nonmelanoma skin cancer (negative control outcome)
Causal contrast	Per-protocol effect, i.e., effect of adhering to assigned strategy on CVD onset	Observational analog of the per-protocol effects
Analysis plan	To estimate the per-protocol effect, we would adjust for pre- and postrandomization factors associated with adherence and loss to follow-up via. IPW Subgroup analyses by baseline age (<60 and ≥60 years old) and sex (males and females).	Same plus additional adjustment for baseline covariates in the outcome regression

^a^The healthy participants should have no prevalent chronic disease. The set of chronic disease we used was cardiovascular disease, diabetes, cancer (apart from nonmelanoma skin cancer), dementia, severe mental diseases (acute stress, phobia, anxiety, depression, schizophrenia, bipolar disorder, and affective disorder), chronic kidney disease, chronic obstructive pulmonary disease, HIV, major inflammatory diseases, Parkinson’s disease, multiple sclerosis, and renal failure.

^b^We considered individuals who had ≥12 clinical consultations or measured their body weight ≥6 times per year as unhealthy.

^c^In the outcome regression models, we adjust for following variables at baseline; age, sex, region (in 8 categories), family history of CVD, BMI. We additionally adjusted for prevalence of hypertension, high LDL measurement before baseline, use of diuretics before baseline, number of weight measurement during the first year (in categories; 1:1 time, 2:2 times, 3:3–5 times), smoking status during the first year (never, former, current), number of clinical consultations during the first year (ordered; 1:≤2 times, 2:3–5 times, 3:6–8 times, and 4:8–11 times).

BMI indicates body mass index; CVD, cardiovascular disease, CHD, coronary heart disease.

#### Protocol of the Target Trial

*Eligibility criteria.* We included individuals aged 45–69 years old, from primary care practices in England registered between 1998 and 2016 without the following chronic diseases: cardiovascular disease, diabetes, cancer (apart from nonmelanoma skin cancer), dementia, severe mental diseases, chronic kidney disease, chronic obstructive pulmonary disease, HIV and major inflammatory diseases.

*Treatment strategies.* Three weight change interventions were applied for 2 years.

(a) loss: ≥3% and <20% of bodyweight each year or undergo bariatric surgery.
(b) weight maintenance: >−3% and <3% of bodyweight change each year.
(c) gain: ≥3% and <20% of bodyweight each year.

These strategies are not prespecified; in other words, body weight may change via. different interventions (e.g., physical activity and diet). We further discuss this point in the Discussion.

*Treatment assignment.* At random

*Follow-up.* 7 years

*Outcomes.* The primary outcome is a composite CVD (CVD death, myocardial infarction, stroke, hospitalization from CHD and heart failure). The secondary outcomes were composite CHD, myocardial infarction, stroke, heart failure, CVD deaths.

*Causal contrasts.* We are interested in the per-protocol effect, that is, the effect of these interventions on CVD, had all individuals adhered to their assigned interventions for both the first and the second year.

*Analysis plan.* To estimate the per-protocol effect of these 2-year interventions, we would first break all the follow-up time of each individual into 1-year periods. We would then have to account for all pre- and postrandomization factors that were related to adherence for the first and the second year, using an inverse probability for treatment weights (IPTW). With this procedure, we weight each observation by the inverse of the probability of an individual having adhered to his or her assigned weight change intervention, given his or her past intervention and prognostic factors history. We explain in detail this procedure in the statistical analysis of the emulated trials.

#### Protocol of the Emulated Trial

*Eligibility criteria*. Same as the target trial plus the following modifications: (i) all participants should have measurements of smoking status (important confounder). (ii) Although the target trial would apply eligibility criteria and assign treatment strategies at a unified time zero, our emulated trial applies the exclusion criteria from chronic diseases based on observing a 12-month baseline period. (iii) We also excluded individuals who had ≥12 clinical consultations or measured their body weight at least six times in the primary care setting during the first year.

*Treatment strategies.* Same as for the target trial

*Treatment assignment.* Individuals are not initially randomized. We classified each individual into the weight loss, maintenance, and gain groups, according to their observed weight trajectories during the first year of follow-up

*Follow-up.* 7 years

*Outcomes.* Same as the target trial plus sensitivity analysis using diabetes as a positive control outcome and nonmelanoma skin cancer as a negative control outcome. We discuss in detail the use of positive and negative control outcome in the sensitivity analysis

*Causal contrasts.* Same as for the target trial

*Analysis plan.* We additionally adjusted for confounders in the baseline period to emulate baseline randomization. See details below.

### Statistical Analysis of the Emulated Trials

Pooled logistic regression models were used to estimate the hazard ratios of the hypothetical interventions and the cumulative incidence risk curves of each intervention,^9^ after dividing the 7-year follow-up time into 1-year periods. Time of entry in the emulated trials was considered the date of the first BMI observations. Each time point corresponds to 1-year duration of our interventions. To emulate randomization in the baseline period, we adjusted for: age (in years), sex (man/woman), BMI (in kg/m^2^), prevalence of hypertension (yes/no), record of high LDL levels (before baseline; yes/no), use of diuretics before baseline (yes/no), family history of CVD (yes/no), hypertension during the first year (yes/no); high LDL levels during the first year (yes/no), use of diuretics during the first year (yes/no), smoking status during the first year (never, former and current), bodyweight measurements during the first year (categorical: 1, 2, and 3–5 measurements), clinical consultations during the first year (categorical: 1–2, 3–5, 6–8, and 9–11 consultations), and region (categorical; London, South West, South Central, South East, East, West Midlands, Central North and North West). Nonadherence occurred when individuals were assigned to a particular weight change group in the first year but deviated from it in the second year. We used IPTW to adjust for time-fixed and time-dependent confounders^9,12^ that were related to adherence in the second year. To calculate the denominator of the IPTW, we used multinomial logistic regression models to model weight loss, maintenance, and gain in the second year, as a function of prognostic factors measured before baseline, during the first and second year of these interventions, along with the observed weight change intervention of the first year. These weights remained unchanged after the second year because we were interested in the effect of interventions sustained over 2 years only. After calculating the IPTW for the received intervention in the second year, individuals were then censored during the second year if they deviate from their assigned intervention. Individuals who developed a chronic disease other than CVD during the second year (and thus were allowed to deviate from their intervention) were assigned the weight of 1 across all time points. We note that we used the nonstabilized IPTW because the regime of the trials was dynamic^12^ (as there are clinically allowable reasons after which individuals were free to deviate from their initial intervention).

We also adjusted for pre- and postrandomization prognostic factors of loss to follow-up through inverse probability of censoring weighting (IPCW) to estimate the effect of the interventions had individuals remained uncensored during the follow-up.^12^ IPTW and IPCW specific to each time point were multiplied to create time-specific weights. The final weight for each individual at a specific time was the cumulative product of his/her time-specific weights up until that time point. We truncated weights >15 (and higher than the 99th percentile of weights) and set them to 15.

We estimated incidence risk curves by fitting the weighted pooled logistic regression models, by additionally including product terms between intervention and follow-up time (linear, squared, and cubic time) to allow for time-varying effects (see eAppendix [Section 3]; http://links.lww.com/EDE/B828). We did not include these product terms in the calculation of hazard ratios, and hence this part of the analysis relied on the proportional hazard assumption. Finally, we used robust variance estimators to calculate 95% confidence intervals (CI) for the hazard ratio estimates and nonparametric bootstrapping from 500 samples to obtain percentile-based 95% CI for the cumulative incidence estimates.

#### Sensitivity Analysis

We used positive and negative control outcomes because any deviation from the expected associations would help us detect potential biases in our emulated trials. In the analysis of the positive control outcome, we expect to observe a nonnull relationship between the exposure and the outcome, although, in the analysis of the negative control outcome, we anticipate estimating no association. We expected that weight loss would be related to lower diabetes risk and weight gain to higher diabetes risk.^24^ We chose nonmelanoma skin cancer as a negative control outcome because there is no established connection between weight change and nonmelanoma skin cancer.

To examine whether results were affected by unaccounted confounders, we redefined the eligibility criteria to individuals by additionally requiring that individuals had measurements of IMD, ethnicity, and physical activity (assuming that last observation carries forward for 4 years). We took these variables into account when adjusting the pooled logistic regression (to emulate randomization) and when estimating the IP weights. We also applied subgroup analysis by age and sex, and we repeated the analysis^1^ using different cutoffs for the weight gain (3%–10% gain per year) and loss (3%–10% loss per year) arms,^2^ after additionally adjusting for calendar year,^3^ and in the obese nonsmokers only.

Moreover, to take into consideration potential preclinical diseases, we assumed that a chronic disease (from the set that was described in the inclusion criteria of the target trial) occurred 1, 2, or 3 years before it was recorded during follow-up and assessed whether estimates were robust to this decision.

## RESULTS

**FIGURE 1.**
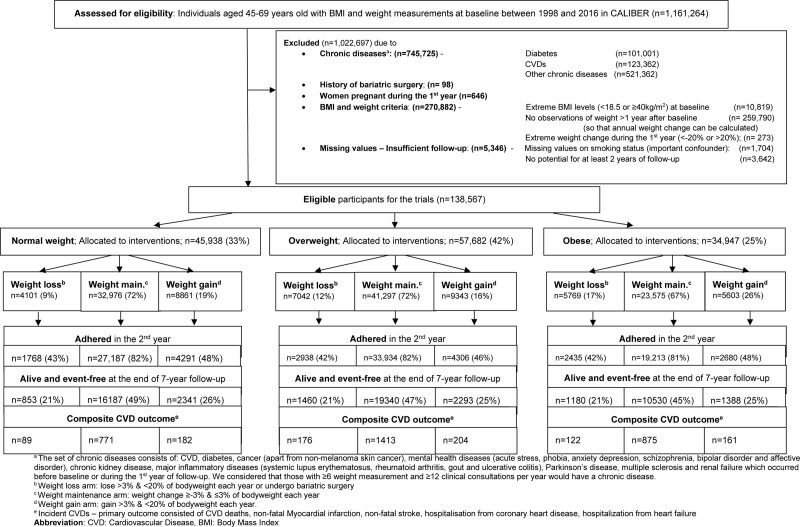
CONSORT diagram—Emulating 2-year weight change interventions in normal weight, overweight, and obese individuals.

Of 1,161,264 individuals aged 45–69, in the primary care database with BMI and weight measurements between 1 January 1998 and 30 May 2016, 138,567 were eligible for the hypothetical trials conducted separately in individuals with normal weight, overweight, and obesity (Figure 1). Specifically, the emulated trials included 45,938 normal weight, 57,682 overweight and 34,947 individuals with obesity. Additionally, in Table 2, we observed that the percentage of individuals who adhered to their assigned intervention during the second year was much higher in the weight maintenance group in all three trials compared with the other arms. In the weight maintenance group, the percentage of women was lower compared with the other two groups. Additionally, individuals in the weight maintenance group had fewer clinical consultations and had their weight measured within primary care less frequently during the first year.

### Emulated Trials in the Normal Weight

**FIGURE 2.**
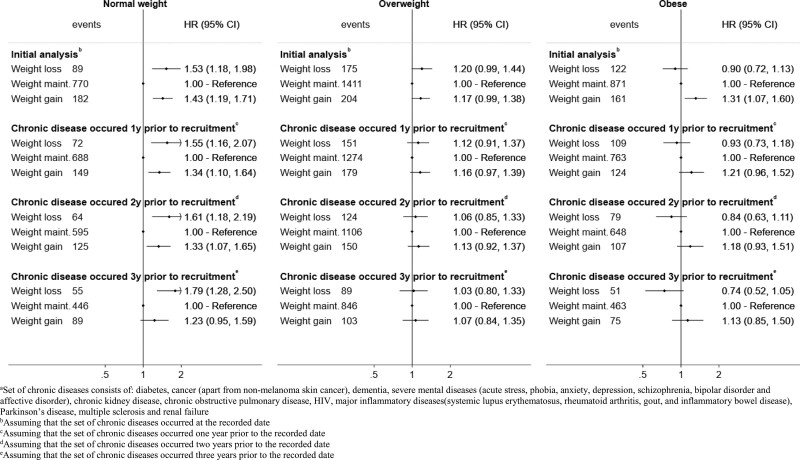
Estimated hazard ratios for cardiovascular diseases comparing hypothetical weight change interventions, by BMI group. Results from initial analysis as well from the sensitivity analysis, in which a set of chronic diseases^a^ was assumed to occur 1, 2, or 3 years before the recorded date.

**FIGURE 3.**
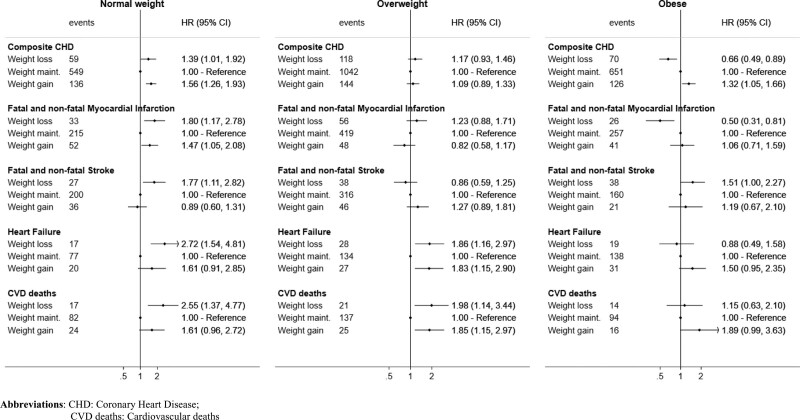
Estimated hazard ratios for cardiovascular diseases (secondary outcomes) comparing hypothetical weight change interventions, using pooled logistic regression.

Among normal-weight individuals, those in the weight loss and weight gain groups had a higher risk for the composite CVD outcome (Figure 2). The risk difference (RD) for weight loss vs. maintenance was 1.5% (0.3% to 3.0%)] and gain versus maintenance was RD = 1.3% (0.5% to 2.2%). Compared with weight maintenance, the hazard ratios for weight loss were 1.53 (1.18–1.98) and for weight gain 1.43 (1.19–1.71). A similar pattern was observed for most of the secondary outcomes (Figure 3). Estimates were similar in subgroups of individuals defined at baseline according to age, but different for females compared to males (HR = 1.72 [1.22–2.41] for men, HR = 1.28 [0.84–1.96] for women, comparing weight loss to weight maintenance) (see eAppendix and eFigure 1; http://links.lww.com/EDE/B828). In sensitivity analyses, estimates were similar with additional adjustment for IMD, ethnicity or physical activity (see eAppendix and eFigure 2; http://links.lww.com/EDE/B828). Estimates were also similar when we assumed that a set of chronic diseases occurred 1, 2, or 3 years before they were recorded in the database (see Figure 2 and eFigure 3; http://links.lww.com/EDE/B828).

### Emulated Trials in the Overweight

**FIGURE 4.**
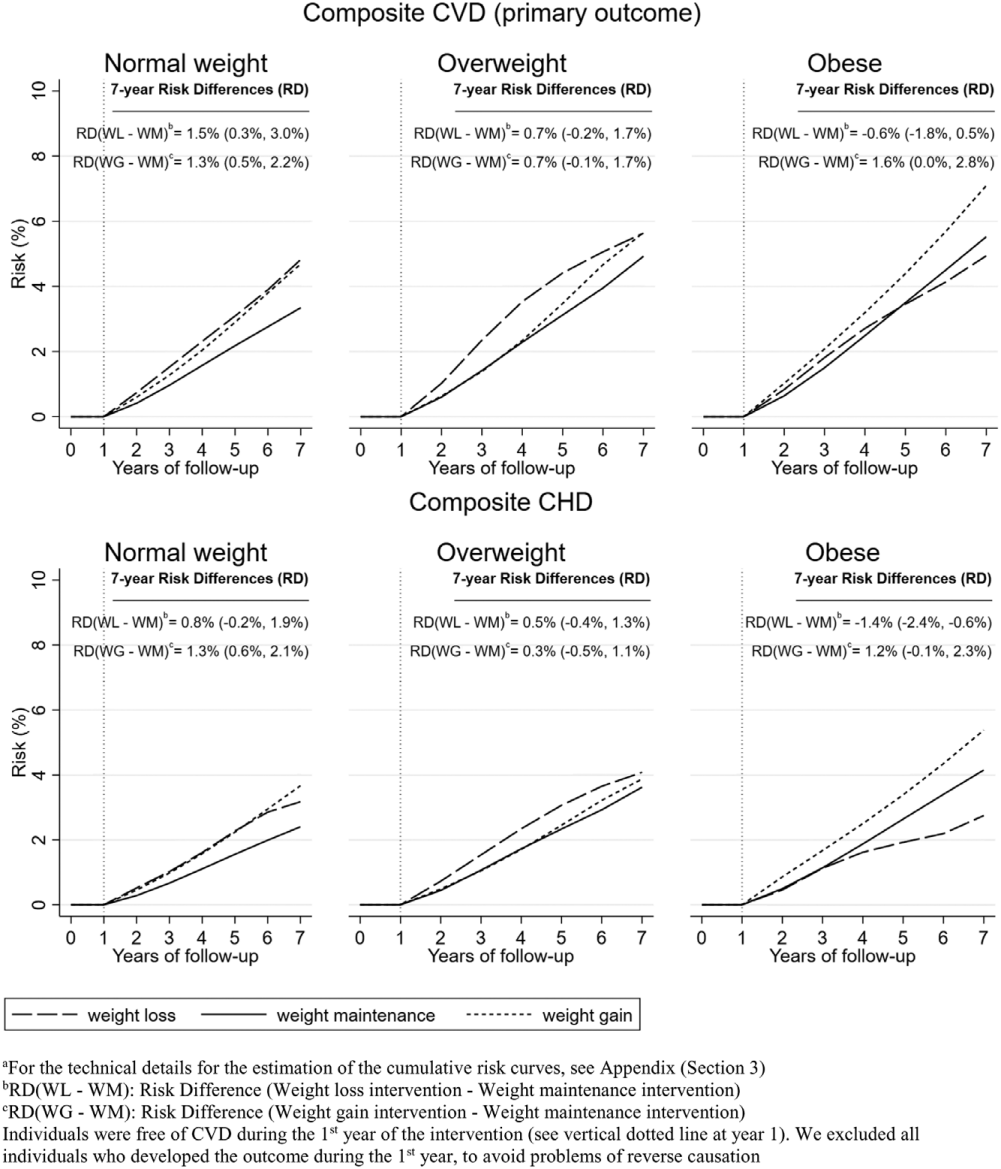
Cumulative incidence curves^a^ for composite CVD and coronary diseases under hypothetical weight change interventions, by BMI group.

Among individuals with overweight, those in the weight loss and weight gain groups had a higher risk for CVD, compared with those in the weight maintenance groups (HR = 1.20 [0.99–1.44] for weight loss and 1.17 [0.99–1.38] for weight gain; Figure 2). In Figure 4, the 7-year RD of CVD for weight loss versus maintenance was 0.7% (−0.2% to 1.7%) and for weight gain versus maintenance was 0.7% (−0.1% to 1.7%). Estimates were similar with additional adjustment for IMD, ethnicity, or physical activity (see eAppendix and eFigure 4; http://links.lww.com/EDE/B828) and in subgroup analyses by age and sex (see eAppendix and eFigure 4; http://links.lww.com/EDE/B828). However, we did observe some differences when considering that chronic diseases occurred 1, 2, or 3 years before the recorded diagnosis date. Estimates for weight loss and weight gain (vs. weight maintenance) were attenuated with increasing lags for chronic disease records (Figure 2 and eAppendix and eFigure 3; http://links.lww.com/EDE/B828).

### Emulated Trials in Individuals With Obesity

Among individuals with obesity, those in the weight gain group had a higher risk for CVD compared with those in the maintenance group (Figure 2; HR = 1.31 [1.07–1.60]). The hazard ratio of CVD for weight loss versus maintenance was 0.90 (0.72–1.13). In the analysis of the secondary outcomes (Figure 3), those in the weight-loss group had a much lower risk of CHD (HR = 0.66 [0.49–0.89]) and myocardial infarction (HR = 0.50 [0.31–0.81]). The estimated 7-year risk of CHD was 2.7% (2.0% to 3.6%) for the weight-loss group, 4.2% (3.9% to 4.6%) for the weight maintenance group and 5.4% (4.3% to 6.5%) for the weight gain group (Figure 4). The RD between weight loss versus maintenance was −1.4% (−2.4% to −0.6%). In contrast, individuals in the weight-loss group had a higher risk for stroke (Figure 3, HR = 1.51 [1.00–2.27]). Estimates were similar with additional adjustment for IMD, ethnicity, or physical activity (eAppendix and eFigure 4; http://links.lww.com/EDE/B828). However, hazard ratios for CVD comparing weight loss to weight maintenance differed by sex (in eFigure 5; http://links.lww.com/EDE/B828, HR = 1.03 [0.79–1.34] in men and HR = 0.65 [0.43–1.01] in women) and age (HR = 0.83 [0.61–1.14] in individuals aged 45–59 and HR = 1.03 [0.73–1.46] in individuals aged 60–69). Moreover, estimated hazard ratios comparing weight loss to weight maintenance decreased with increasing lags for chronic disease records (HR = 0.93 [0.73–1.18] under 1-year lag, 0.84 [0.63–1.11] under 2-year lag, and 0.74 [0.52–1.05] under 3-year lag; Figure 2).

### Positive and Negative Control Outcomes

In analyses of diabetes as a positive control outcome, the cumulative risk was higher in the weight-loss group than in weight maintenance group during the first 2–3 years, in all BMI groups (eFigure 6; http://links.lww.com/EDE/B828). The corresponding hazard ratios of weight loss (vs weight maintenance) were, across BMI groups, positive up to the first 3 years [HR = 1.39 (0.96–2.00), HR = 1.53 (1.27–1.93) and HR = 1.32 (1.13–1.54) in the normal weight, overweight, and individuals with obesity respectively], and negative after the third year [HR = 0.76 (0.38–1.53), HR = 0.88 (0.60–1.28), and HR = 0.64 (0.46–0.88) in the normal weight, overweight and individuals with obesity, respectively]. In analyses of nonmelanoma skin cancer as a negative outcome control, the hazard ratios of both weight gain and weight loss were close to one (eFigure 7; http://links.lww.com/EDE/B828).

### Other Sensitivity Analyses

Results remained largely the same when we reanalyzed the data^1^ using different cutoffs for the weight gain (3–10% gain per year) and loss (3–10% loss per year) arms (eFigure 8; http://links.lww.com/EDE/B828)^2^ and after additionally adjusting for calendar year (eFigure 9; http://links.lww.com/EDE/B828). Moreover, the results in the obese nonsmokers (eFigure 10; http://links.lww.com/EDE/B828) were similar to the ones in the obese overall; nevertheless, this analysis was less powered due to the reduced sample size.

## DISCUSSION

In this study, we used large-scale EHR data to emulate target trials of weight loss, maintenance and gain interventions, estimating their effect on incident CVD. We found that, among normal-weight individuals, the weight maintenance group had a lower CVD risk compared with the weight gain and the weight-loss group. Among individuals with overweight, the weight loss and weight gain groups had a slightly higher CVD risk compared with weight maintenance; however, this finding was not robust in sensitivity analyses for unmeasured confounding by preclinical diseases. Among individuals with obesity, the weight-loss group had a lower risk of CHD but not for stroke, heart failure, and CVD deaths.

### Strengths and Limitations

Key strengths of this study include the use of large-scale EHR and the application of cutting-edge causal inference methods,^9-12^ the combination of which gave us the opportunity to emulate weight change trials in 138,567 otherwise healthy individuals by BMI group. Such large target trials would be impossible to conduct, as most of the actual trials recruit people with chronic disease and do not follow them up for sufficient time to measure hard outcomes, like CVD.^3^ Another advantage of this paper is that in previous studies focusing on the effect of BMI change, not in the trial emulation framework, different methodologies have been used to define baseline and then to adjust for baseline confounders. We present in another paper in detail why the definition of a baseline period^25^ minimizes the risk of inappropriate nonadjustment for confounders and why the trial emulation framework is the optimal way of estimating the relationship between a risk factor’s change and a health outcome. Moreover, the positive and the negative control outcome were very important to find potential problems in the confounding structure of the data. In our case, the paradoxical findings of the positive control outcome (i.e., that weight loss is a risk factor for diabetes in the first 3 years) were explained by reverse causation (actual date of diabetes was before the recorded date) and lead our sensitivity analysis, in which we assumed that the actual date of chronic diseases was 1, 2, and 3 years before the recorded date.

Several limitations should be noted. The observational study design of this research, despite our use of causal inference methods, is still prone to unmeasured confounding. We tried to observe to what extent the bias due to preclinical diseases would affect our results by emulating the same interventions using positive and negative control outcomes. We observed that weight loss was related to higher risk for diabetes, compared with weight maintenance, during the first 2–3 years. This unexpected result may be due to unmeasured confounding by subclinical disease (i.e., reverse causation). This was an indication that individuals who developed a chronic disease during the first 3 years of follow-up might not had been healthy at baseline. To address this problem, we considered that the actual date of the occurrence of a chronic condition was 1, 2, or 3 years before the recorded date and observed whether there was any specific trend in our findings from this sensitivity analysis. Moreover, it is important to mention that we could not identify the way different people lost, maintained, or gained weight (e.g., diet, physical activity), as our hypothetical interventions are not well defined. Different methods of modifying bodyweight may have different effects on CVD risk.^26^ Ill-defined interventions also affect our ability to define and address confounding too. Our results can be viewed as estimates for an effect of a weighted average of several specific strategies that result in bodyweight change with (unknown) weights reflecting their frequency in our population.^27^ Furthermore, we could only identify at which arm individuals were allocated only at the end of the first year, based on their observed weight trajectory (Appendix, Section 1; http://links.lww.com/EDE/B828). In this study, as well as in similar hypothetical interventions,^17,18^ each time point corresponds to a specific time period (1 year in our study, that is baseline, is the baseline period between recruitment and the end of the first year). For more details, see Appendix; http://links.lww.com/EDE/B828, Section 6, as well as in our new paper, in which we explain how to estimate a risk factor’s change on a health outcome.^25^ Moreover, we assumed that (a) the last observation carried forward^9^ for at most 4 years for smoking status and (b) there was a linear trend for weight change between two bodyweight measurements recorded less than 4 years apart. Additionally, we could not identify whether weight change was intentional and we did not use a sequential nested structure adding other calendar years to recruit the same patients in the future^9^ to avoid further computational complexities, as our sample size was large. Another limitation was that the weight loss and gain categories were rather broad (3–20% of annual weight loss and gain, respectively); however, when we tightened the cutoffs (3–10%), results were practically unchanged. Finally, there might be a risk of extending inferences to the general healthy population^28^ due to selection bias because many people do not measure their bodyweight frequently and thus were excluded from the study.

#### Meaning of the Study: Possible Explanations and Implications

In our study, the average (SD) percentage of weight change during the first year in the weight-loss arm was −5.3% (2.3%) in the normal weight, −5.5% (2.6%) in the overweight, and −6.1% (3.1%) in the obese. In the United Kingdom, the NHS weight-loss guide includes increased levels of physical activity and a calorie limit of no more than 1,900 kcal a day for most men and 1,400 kcal for most women.^29^

First, we observed that weight maintenance, compared with weight loss or gain, was linked with lower risk for all the CVD endpoints in normal-weight individuals. These results were in line with the cohort studies that have shown that normal-weight individuals have less risk for developing CVD^7^ compared with the other BMI group and thus infer that these individuals should not gain or lose weight.

Among individuals with overweight, we initially found evidence consistent with the hypothesis that both weight gain and loss moderately increases CVD risk; however, estimates were attenuated (especially for weight loss) in sensitivity analyses when we assumed that a chronic disease might have occurred before the recorded date. These findings are similar to those of two previous studies.^18,19^ In the first study,^18^ overweight and obese individuals were merged together, although in the second,^19^ the authors’ inability to capture a protective association might have been due to the moderate sample size.

In people with obesity, weight gain was related to a higher risk for CVD, which is in line with the numerous reports that have linked high BMI to the onset of CVD.^7^ Weight loss was not related to a clear CVD risk reduction, even though in the sensitivity analysis, we observed that the more years, we assumed the set of chronic diseases happened before the recorded date, the lower the hazard ratio for CVD of the weight loss was. When we stratified the analysis by age and sex, we found that lower risk of CVD for women and younger individuals. Furthermore, weight loss was related to lower risk for CHD and especially to fatal and nonfatal myocardial infarction. Individuals with obesity should reduce their body weight to lower their risk for CHD. The US Preventive Services Task Force currently recommends behavioral weight-loss interventions for individuals with obesity (BMI ≥ 30 kg/m^2^).^30^ Nevertheless, the expected beneficial effect from weight reduction was only detectable for CHD, but not for stroke, in our study. Although the American Stroke Association recommends weight loss for people with overweight or obesity for the primary prevention of ischemic stroke,^31^ we did not estimate any benefit from weight reduction. This may be due to the fact that BMI is an imprecise measure of body fat and does not distinguish between fat and muscle mass.

### Unanswered Questions and Future Research

Our study could not clarify how individuals lost, maintained, or gained weight. More studies should focus on how to emulate trials from observational data to investigate how adherence to different diets or physical activity levels that result in weight change could affect a range of CVD outcomes.

## CONCLUSIONS

Individuals who maintained their weight had the lowest risk for CVD among those with normal weight. Among individuals with obesity, the weight-loss group had a lower risk of CHD. Weight gain was associated with increased risk of CVD across BMI groups. The paradoxical findings that weight loss was associated with increased risk for diabetes (positive control outcome) in the first 3 years [HR = 1.39 (0.96–2.00), HR = 1.53 (1.27–1.93), and HR = 1.32 (1.13–1.54) in the normal weight, overweight, and individuals with obesity, respectively] guided our sensitivity analysis in which we assumed that the actual date of chronic diseases was 1, 2, and 3 years before the recorded date. Our results may help to inform policy guidelines for cardiovascular prevention.

**TABLE 2.** Characteristics of Individuals at Baseline and During the First Year, by BMI Group and Hypothetical Weight Change Intervention

	**A. Intervention in Normal-weight Individuals (N = 45,938**)			**B. Intervention in Overweight Individuals (N = 57,682**)			**C. Intervention in Obese Individuals (N = 34,947**)		
	**Weight loss N = 4,101**	**Weight maintenance N = 32,976**	**Weight gain N = 8,861**	**Weight loss N = 7,042**	**Weight maintenance N = 41,297**	**Weight gain N = 9,343**	**Weight loss N = 5,769**	**Weight maintenance N = 23,575**	**Weight gain N = 5,603**
**Age in years; mean (SD**)	56.2 (7.2)	55.5 (7.0)	54.8 (6.9)	56.4 (7.0)	55.9 (7.0)	55.1 (6.9)	55,6 (6,9)	55.3 (6.8)	54.5 (6.7)
**Sex % of females**	64%	62%	65%	50%	41%	51%	52%	46%	52%
**BMI in kg/m^2^; mean (SD)**	22.8 (1.6)	22.8 (1.6)	22.6 (1.6)	27.4 (1.4)	27.2 (1.4)	27.2 (1.4)	33.4 (2.6)	33.1 (2.5)	33.2 (2.5)
**Prevalence of hypertensives; %**	26%	23%	21%	32%	30%	27%	37%	34%	31%
**Record of high LDL levels (before baseline**)^a^	6%	6%	5%	10%	9%	8%	10%	9%	8%
**Use of diuretics (before baseline); %**	10%	8%	10%	14%	12%	14%	20%	18%	19%
**Family history of CVD; %**	13%	13%	13%	13%	13%	13%	12%	12%	12%
**Weight change (during the first year) mean (SD**)	−5.3% (2.3%)	0.3% (1.4%)	5.6% (2.8%)	−5.5% (2.6%)	0.2% (1.5%)	5.4 (2.6)	−6.1% (3.1%)	0.1% (1.5%)	5.3% (2.5%)
**Hypertensive (during the first year); %**	17%	15%	15%	24%	21%	19%	29%	26%	23%
**High LDL levels**^a^**(during the first year); %**	6%	5%	4%	9%	7%	6%	9%	7%	6%
**Use of diuretics (during the first year); %**	9%	7%	9%	13%	12%	14%	19%	18%	19%
**Smoking status (during the first year**)									
Never	53%	59%	54%	53%	55%	51%	53%	54%	51%
Former	22%	22%	23%	29%	29%	29%	32%	32%	31%
Current	26%	19%	24%	18%	16%	20%	15%	15%	18%
**Weight measurements (during the first year**)									
1 meas., %	66%	85%	72%	61%	82%	70%	52%	74%	63%
2 meas., %	26%	13%	22%	28%	14%	24%	30%	19%	28%
3–5 meas., %	8%	3%	6%	11%	4%	6%	18%	7%	9%
**Clinical consultations (during the first year**)									
1–2 consultations, %	24%	29%	26%	21%	27%	23%	17%	23%	21%
3–5 consultations, %	34%	38%	37%	36%	36%	36%	35%	36%	34%
6–8 consultations, %	25%	22%	24%	27%	24%	26%	29%	26%	27%
9–11 consultations, %	17%	11%	14%	16%	13%	15%	18%	15%	17%

^a^Individuals with previous LDL records >4.1 mmol/L (high LDL).

## ACKNOWLEDGMENTS

We would like to thank Miguel Hernán and Barbra Dickerman for the discussions and their useful suggestions during the drafting of the article. This study was approved by the Medicines and Healthcare Products Regulatory Agency Independent Scientific Advisory Committee protocol references: 18_010. This study is based in part on data from the Clinical Practice Research Datalink obtained under license from the UK Medicines and Healthcare products Regulatory Agency. The data are provided by patients and collected by the NHS as part of their care and support. The interpretation and conclusions contained in this study are those of the author(s) alone. Hospital Episode Statistics Copyright (2019) is reused with the permission of The Health & Social Care Information Centre. All rights reserved. The Office of Population Censuses and Surveys Classification of Interventions and Procedures, codes, terms, and text is Crown copyright (2016) published by Health and Social Care Information Centre, also known as NHS Digital and licensed under the Open Government Licence (available at www.nationalarchives.gov.uk/doc/open-government-licence/open-government-licence.htm). This study was carried out as part of the CALIBER programme (https://www.ucl.ac.uk/health-informatics/caliber). CALIBER, led from the UCL Institute of Health Informatics, is a research resource consisting of linked electronic health records phenotypes, methods and tools, specialized infrastructure, and training and support. The views expressed are those of the author(s) and not necessarily those of the National Health Service, the National Institute for Health Research, or the Department of Health. This paper represents independent research [part] funded by the National Institute for Health Research (NIHR) Biomedical Research Centre at UCL Hospitals. The views expressed are those of the author(s) and not necessarily those of the NHS, the NIHR or the Department of Health and Social Care.

## Supplementary Material


